# Differential prognostic impact of platelet-derived growth factor receptor expression in NSCLC

**DOI:** 10.1038/s41598-019-46510-3

**Published:** 2019-07-15

**Authors:** Thomas Karsten Kilvaer, Mehrdad Rakaee, Turid Hellevik, Jørg Vik, Luigi De Petris, Tom Donnem, Carina Strell, Arne Ostman, Lill-Tove Rasmussen Busund, Inigo Martinez-Zubiaurre

**Affiliations:** 1 Department of Oncology, University Hospital of North Norway, Tromso, Norway; 2 Institute of Clinical Medicine, UiT The Arctic University of Norway, Tromso, Norway; 3 Institute of Medical Biology, UiT The Arctic University of Norway, Tromso, Norway; 4 Department of Oncology-Pathology Cancer Center Karolinska, Karolinska Institutet, Stockholm, Sweden; 5 Department of Clinical Pathology, University Hospital of North Norway, Tromso, Norway

**Keywords:** Non-small-cell lung cancer, Biomarkers, Prognostic markers

## Abstract

Preclinical evidence suggests that stromal expression of platelet-derived growth factor receptors (PDGFRs) stimulates tumor development and diminishes intratumoral drug uptake. In non-small cell lung cancer (NSCLC), the clinical relevance of stromal PDGFR expression remains uncertain. Tumor specimens from 553 patients with primary operable stage I-IIIB NSCLC was obtained and tissue micro-arrays (TMA) were constructed (Norwegian cohort). Immunohistochemistry (IHC) was used to evaluate the expression of PDGFRα and -β in stromal cells and to explore their impact on patient survival. Results were validated in a non-related cohort consisting of TMAs of 367 stage I (A and B) NSCLC patients (Swedish cohort). High stromal PDGFRα expression was an independent predictor of increased survival in the overall populations and SCC (squamous cell carcinoma) subgroups of both investigated cohorts. PDGFRβ was an independent predictor of poor survival in the overall Norwegian cohort and an independent predictor of increased survival in the ADC (adenocarcinoma) subgroup of the Swedish cohort. Tumors displaying the combination PDGFRα-low/PDGFRβ-high exhibited inferior survival according to increasing stage in the Norwegian cohort. This study confirms that high stromal expression of PDGFRα is a predictor of increased survival in NSCLC. Further exploration of the prognostic impact of PDGFRβ and the relationship between PDGFRα and -β is warranted.

## Introduction

In solid neoplasms, a dynamic relationship between the malignant component and the surrounding stroma is established early during tumorigenesis and is ever evolving during tumor progression. A growing amount of evidence indicate that the tumor microenvironment (TME) affects the growth of tumors in multiple ways at all stages, and has a direct and profound influence on aspects such as tumor cell survival, local invasion, metastatic dissemination and response to therapy^1,2^.

The PDGF/PDGFR axis is one of the best-described tumor-stroma interconnections. Platelet-derived growth factors (PDGF) are strong mitogenic and chemotactic factors for mesenchymal cells such as vascular smooth muscle cells, connective tissue fibroblasts, glomerular mesangial cells, pericytes and neurons^3^. Briefly, the PDGFs are a family of dimeric disulfide-bound growth factors, consisting of four proteins forming five possible dimers *in vivo*, namely PDGF-AA, PDGF-AB, PDGF-BB, PDGF-CC, and PDGF-DD. Each of these isoforms exerts its biological effects by activating two structurally related α- and β-tyrosine kinase receptors. PDGF-AA, PDGF-AB, PDGF-BB, PDGF-CC dimers bind with high affinity to the α-receptor whereas PDGF-BB and PDGF-DD has preference for the β-receptor^4,5^. The three known dimeric PDGF receptor combinations, PDGFR-αα, PDGFR-αβ, and PDGFR-ββ, transduce overlapping but not identical cellular signals^3^. Thus, the net effect of PDGF dimers on cells will depend in the specific expression of each PDGF receptor isoform.

In cancer, PDGFRs are emerging as key regulators of mesenchymal cell activity in the TME^6^. Activation via the PDGF/PDGFR axis may directly impact important tumor biological features such as proliferation, vascular reorganization, endothelial cell activation, pericyte recruitment, regulation of the tumor interstitial fluid pressure and desmoplastic reactions^6^. In malignancies of the breast, colon, pancreas and prostate, high stromal expression of PDGFRβ has been associated with poor prognosis^7–9^. However, the overall prognostic relevance of PDGFRs expression in tumors of epithelial origin is inconclusive due to a substantial number of conflicting reports^6^. Still, the clinical relevance of PDGFRs has been reinforced through studies leading to approval of drugs with PDGFR-inhibitory activity^10^. In the particular case of non-small cell lung cancer (NSCLC) patients, several new agents that involve directly or indirectly blocking of the PDGFR signaling, e. g., linifanib, motesanib and olaratumab, are being tested (Clinical trilas.gov). In a previous study by our group, PDGFRs were evaluated along with their cognate ligands, in both tumor-cells and stroma of 335 NSCLC patients^11^. High expression of PDGFRα in tumor cells, was identified as an independent indicator of poor disease-specific survival (DSS), while high expression of PDGFRα in stromal cells, was found to be a significant, but not independent, indicator of increased DSS. However, in this study, evaluation of stromal expression did not distinguish between expression in fibroblasts (spindle shaped cells) and spurious expression in other cell types such as immune cells^11^. Hence, this study focuses on the association of PDGFRα and -β expression in cancer-associated fibroblasts and patients prognosis in tissue from 553 stage I-IIIB NSCLC patients. An independent cohort of 367 stage I (A and B) NSCLC patients is used for validation of results.

## Materials and Methods

### Patient cohort

A summary of the patient cohorts is given in Table 1. Briefly, the Norwegian population consisted of an unselected population of 553 patients diagnosed with stage I-IIIB NSCLC at the University Hospital of North-Norway from 1990–2010. The cohort is extensively documented^11–13^. The Norwegian cohort has been revised according to the latest 2015 WHO guidelines on histological classification and 8^th^ edition of the UICC guidelines on staging of lung tumors, as previously described by Hald *et al*.^13,14^. The validation cohort (Swedish cohort) consisted of 367 patients diagnosed with stage I (A and B) NSCLC at Karolinska University Hospital from 1987–2002. The cohort has previously been documented^15–17^. The Swedish cohort has been revised according to the 2004 WHO guidelines on histological classification and staged after the 7^th^ edition of the UICC guidelines on staging of lung tumors^18^.

**Table 1:** Summary and comparison of clincopathological and technical characteristics for (A) The Norwegian cohort and (B) The Swedish cohort.

	(A) Norwegian cohort	(B) Swedish cohort
Number of patients	553	367
SCC	307	109
ADC	239	209
Other	7	49
Time of inclusion	1990–2010	1987–2002
Median age in years	67 (28–85)	68 (41–86)
Date of last follow-up	2013-10-01	2010-06-30
Median follow-up of survivors (months)	86 (34–267)	122 (28–122)
Available clinical data	Age, gender, smoking status, ECOG PS, weightloss before diagnosis, surgical procedure, adjuvant radiotherapy and/or chemotherapy	Age, gender, smoking status, surgical procedure, adjuvant radiotherapy and/or chemotherapy
Available pathological data	Histology, differentiation, pStage, tStage, nStage, resection margins, vascular invasion, perineural infiltration	Histology, pStage, tStage, nStage, resection margins
Available endpoints	OS, DSS, PFS	OS
TMA core size	0.6 mm	1 mm
Number of TMA cores for each patient	Four – two primarily stromal and two primarily epithelial	Two – primarily epithelial
Slice thickness	4 µm	4 µm
Distribution of scores		
PDGFRα	Low 366/High 152/Missing 35	Low 232/High 113/Missing 22
PDGFRβ	Low 311/High 202/Missing 40	Low 208/High 134/Missing 25

Abbreviations: SCC, squamous cell carcinoma; ADC, adenocarcinoma; TMA, tissue micro-array; PDGFR, platelet-derived growth factor receptor; OS, overall survival; DSS, disease-specific survival; PFS, progression-free survival.

The Regional Committee for Medical and Health Research Ethics (REK-Nord) and the Institutional Review Boards at Karolinska Institutet and at Stockholms County Council approved the use of human material for the Norwegian (Project-ID: 2016/2307/REK-Nord) and Swedish cohorts, respectively. Due to the retrospective nature of the study, and the fact that two thirds of the study population was deceased at time of study initiation, the need of written informed consent was waivered. All methods involving human material were performed in accordance with relevant guidelines and regulations.

### Tissue micro-array construction

Tissue micro-arrays were constructed according to standard procedures previously described^19^. Representative areas were identified on H&E slides of primary lung cancer patients, by an experienced pathologist. The TMA cores were sampled using the marked H&E slides as overlay. In the Norwegian cohort, four 0.6 mm cores, two from tumor epithelial and two from stromal areas were sampled for each patient. In the Swedish cohort, two 1.0 mm cores from tumor epithelial areas were sampled for each patient. TMA blocks were cut into 4μm sections and stained for PDGFRα, and -β.

### Immunohistochemistry

The staining procedures were previously described^20^. Briefly, the immunohistochemical staining for both cohorts was performed using the Discovery-Ultra platform (Ventana, Roche). After on-board de-paraffinization and antigen retrieval (Cell conditioning 1 solution, 48 min), the following rabbit monoclonal primary antibodies were applied: PDGFRα (cell signaling, Cat #5241, clone: D13C6, dilution, 1/100); PDGFR- β (cell signaling, Cat #3169, clone: 28E1, dilution:1/50). The secondary antibody was ﻿UltraMap anti-rabbit horseradish peroxidase (Ventana, Cat:# 760-151), which was incubated for 20 minutes, followed by 12 minutes of amplification using the HQ-HRP amplification kit (Ventana, Cat:#760-052). The immune reaction signals were detected by Discovery Chromomap DAB kit (Ventana, Cat:#760-159). Finally, the slides were counter-stained by hematoxylin II (Ventana, Cat: #790-2208) for 28 minutes and then a bluing reagent (Ventana, Cat:#60-2037) for 4 minutes.

*Antibody validation* To ensure staining specificity, an isoptype-matched control antibody was used. Multiple organ TMA containing positive and negative tissue controls was used to further verify the specificity of every staining procedure. In addition, IHC was conducted with specific antibodies previously validated using formalin-fixed paraffin-embedded preparations of cultured cells with known PDGFRα and -β status^6^.

### Scoring of IHC

**Figure 1:**
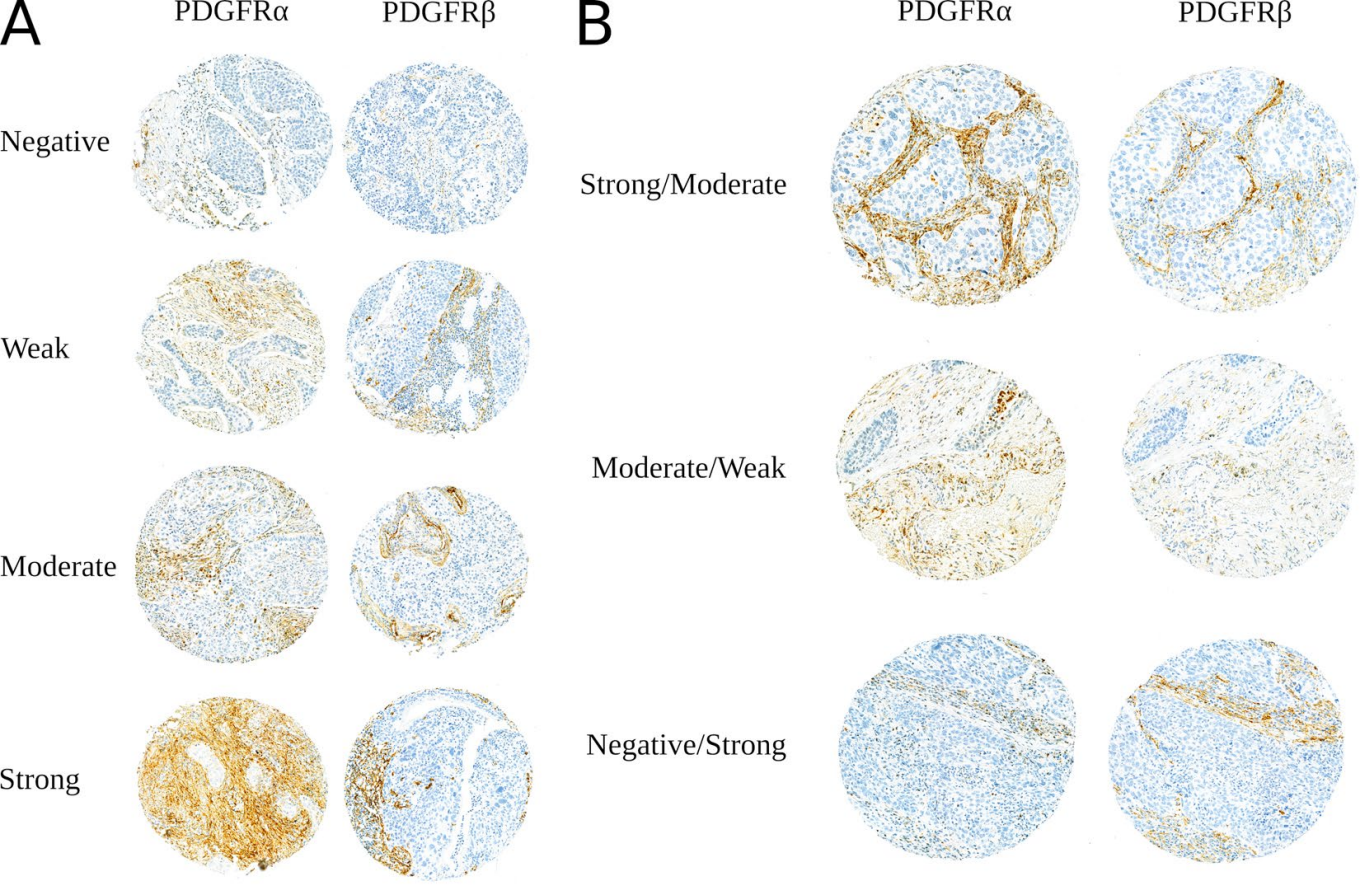
(**A**) Examples of TMA cores exhibiting negative, low, moderate and high expression of PDGFRα and PDGFRβ. (**B**) Consecutive cores showing different scores for PDGFRα and PDGFRβ. Areas with PDGFR expression clearly overlap in some cores while no overlap is observed for other cores. Abbreviations: PDGFR, platelet-derived growth factor receptor.

TMAs from the Norwegian cohort were reviewed using a Leica DM 2500 microscope (Leica Microsystems). TMAs from the Swedish cohort was reviewed on computer screen after digitalization on a 3DHistech Pannoramic Flash III (3DHistech). After initial review a semi-quantitative score was established. The dominant staining intensity in tumor-associated stroma was scored as follows: 0 = no, 1 = weak, 2 = moderate, 3 = strong, using the same scale for both PDGFRs (examples in Fig. 1A). Staining was evaluated specifically in spindle-shaped stromal cells. The two most representative TMA spots were assessed by two independent scorers, resulting in four scores for each patient. Cut-offs were chosen using a minimal P-value approach yielding low/high groups of comparable size between the cohorts, for both markers.

### Statistical methods

All statistical analyses were conducted in RStudio version 1.1.456 with R version 3.5.1 and packages “Hmisc”, “reshape2”, “sjmisc”, “survival”, “ggplot2”, “plyr”, “grid”, “gridExtra”, “irr”, “gdata” and “cowplot”. Between-scorer agreement was assessed by 1) a two-way random-effects model with absolute agreement definition and 2) Cohen’s kappa-statistics with equal weights. Cohen’s Kappas and the intraclass correlation coefficients were obtained from these results. Associations between dichotomized markers and clinicopathological variables were tested with Chi-square or Fisher’s Exact tests. The log-rank test and the Kaplan-Meier method was used to assess the difference between and to visualize survival curves. For the Norwegian cohort OS, DSS and PFS were available end-points. In this cohort, OS was defined as the time from surgical resection to death of any cause, DSS was defined as the time from surgical resection to lung cancer specific death and PFS was defined as the time from surgical resection to first metastasis or first local recurrence. In the Swedish cohort, OS was the only available end-point. In this cohort OS was defined as the time from surgical resection to death of any cause. Patients living 10 years or longer were censored in the Swedish cohort. A supervised iterative process was used to fit multi-variable cox proportional hazard models to data in order to investigate markers in the presence of each other and other clinicopathological variables.

For all statistical tests a significance level below 0.05 was deemed statistically significant.

## Results

### Clinicopathological variables

Clinicopathological variables for both the Norwegian and Swedish cohorts are summarized in Table 1 and visualized across PDGFR expression in Table 2. Age at diagnosis and distribution of gender and smoking status were comparable for the two cohorts. Distribution of histological subgroups were not comparable between the cohorts with 56% and 30% in the SCC subgroups and 43% and 57% in the ADC subgroups, in the Norwegian and the Swedish cohorts, respectively (Table 1).

**Table 2:** Correlations between clinicopathological variables and PDGFRα and-β in the (A) Norwegian cohort and (B) Swedish cohort (chi-square and Fisher’s exact tests)

	(A) Norwegian cohort						(B) Swedish cohort					
	PDGFRα			PDGFRβ			PDGFRα			PDGFRβ		
	Low	High	P	Low	High	P	Low	High	P	Low	High	P
Age			0.380			0.390			0.694			0.254
<65	149	69		127	91		98	51		95	52	
≥65	217	83		184	111		134	62		113	82	
Gender			0.330			0.780			0.575			0.449
Female	117	56		105	65		106	56		100	58	
Male	249	96		206	137		126	57		108	76	
Weightloss			0.630			0.100						
<10%	331	135		285	176							
>10%	34	17		25	26							
Smoking			0.650			0.130			0.595			0.200
Never	13	4		14	3		18	8		14	11	
Present	227	101		190	134		121	65		121	63	
Previous	126	47		107	65		65	27		53	40	
Unknown							28	12		20	19	
ECOG PS			0.740			**<0.001**						
0	213	94		202	101							
1	126	48		91	82							
2	27	10		18	19							
Histology			0.230			**0.010**			0.720			0.975
SCC	204	85		163	123		70	32		60	42	
ADC	158	64		146	74		134	64		118	76	
LCC	3	0		1	2		4	1		4	2	
ASC	1	2		0	3		18	10		18	10	
NOS	0	1		1	0		6	6		8	4	
Tstage			0.180			0.740			0.332			0.804
T1a	9	5		7	6		80	33		70	42	
T1b	47	19		44	22		78	33		62	46	
T1c	72	19		57	33		43	30		46	26	
T2a	88	31		72	45		31	15		30	18	
T2b	49	22		38	32							
T3	60	39		61	38							
T4	41	17		32	26							
Nstage			0.270			0.960						
N0	249	107		211	139							
N1	85	27		68	42							
N2	32	18		32	21							
Pstage			0.720			0.310			0.146			0.627
IA1	6	3		3	5		158	66		132	88	
IA2	41	17		37	21							
IA3	56	15		45	24							
IB	54	21		51	24		74	45		76	44	
IIA	29	16		21	23							
IIB	95	38		77	52							
IIIA	73	37		64	47							
IIIB	12	5		13	6							
Differentiation			0.090			0.590						
Poor	154	59		131	78							
Moderate	152	77		138	91							
Well	60	16		42	33							
Vascular invasion			0.440			1.000						
No	304	122		254	166							
Yes	60	29		55	35							

Abbreviations: PDGFR. Platelet-derived growth factor receptor; ECOG PS, Eastern Cooperative Oncology Group performance status; ADC, adenocarcinoma; SCC, squamous cell carcinoma; LCC, large-cell carcinoma; ASC, adenosquamous carcinoma; NOS, not otherwise specified; Tstage, tumor stage; Nstage, nodal stage; Pstage, pathological stage.

### Interobserver reliability

For both the Norwegian and the Swedish cohorts between scorer agreement was sufficient. In the Norwegian cohort, ICC and kappa was 0.92 and 0.92 and 0.73 and 0.75 for stromal PDGFRα and PDGFRβ, respectively. In the Swedish cohort, ICC and kappa was 0.90 and 0.88 and 0.68 and 0.66 for stromal PDGFRα and PDGFRβ, respectively

### Expression of PDGFRs and their correlations

Expression of PDGFRs serial cores are visualized in Fig. 1B. In the stromal compartment, PDGFRα was expressed in fibroblasts, vessel-like structures and in some few cases round-shaped immune cells. In addition, PDGFRα was, to some extent, expressed in the tumor epithelial-cells of 18% of the patients (20% of SCCs and 16% of ADCs) in the Norwegian cohort. Expression in tumor was not evaluated in the Swedish cohort. PDGFRβ was exclusively expressed in fibroblasts and vessel-like structures. As illustrated in Fig. 1B, patterns of staining of the two receptors in serial sections were overlapping in some, but not all cores. It is likely that some cells co-express the two PDGFRs.

Table 2 summarizes the associations between low and high expression of PDGFRα and -β and clinicopathological variables for both the Norwegian and Swedish cohorts. No associations were observed for variables available in both cohorts. In the Norwegian cohort, high expression of PDGFRβ was associated with ECOG PS (P < 0.001).

### Survival analyses

#### Univariate analyses

**Figure 2:**
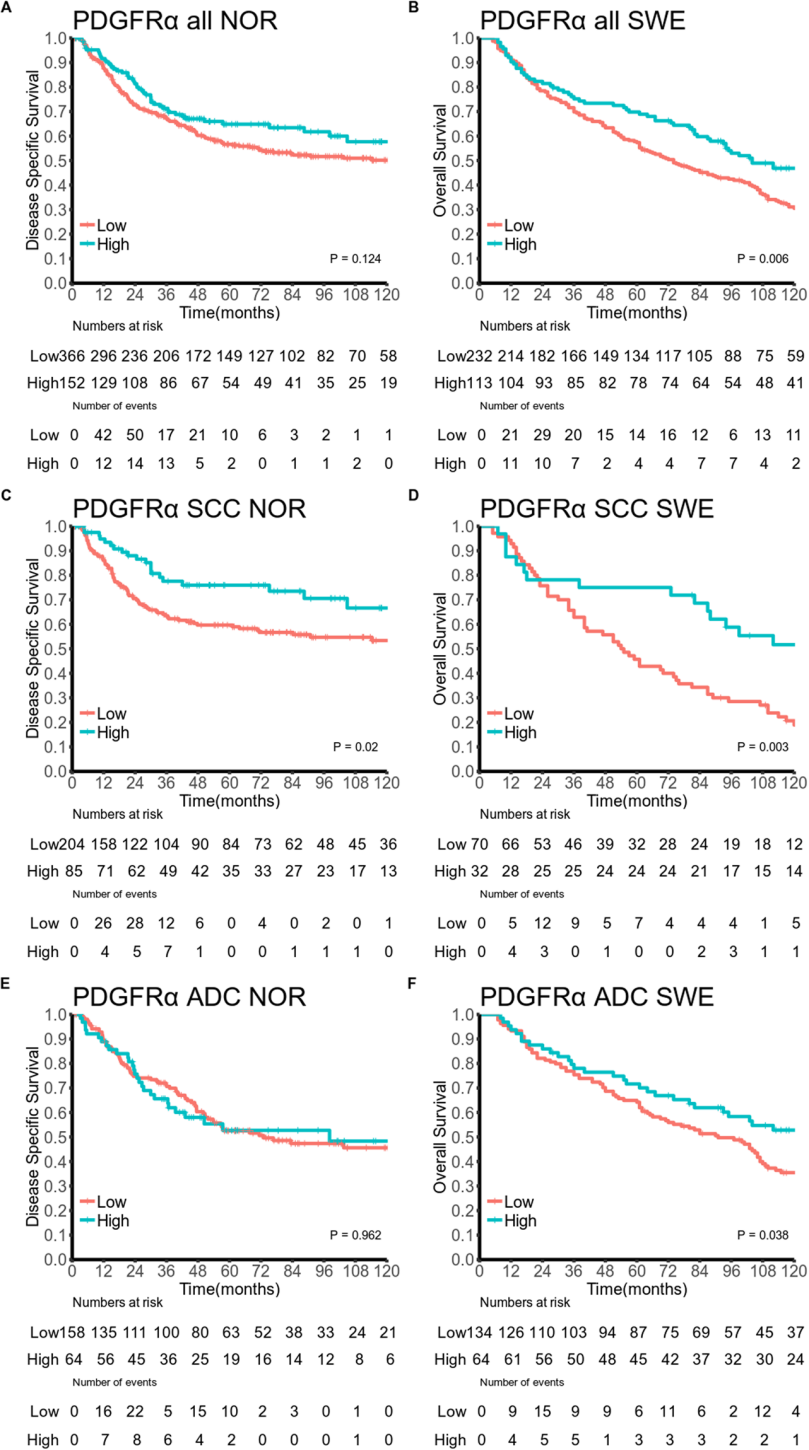
Survival curves for PDGFRα expression in the overall cohorts and in the SCC and ADC subgroups for the Norwegian cohort (**A**,**C**,**E**) and the Swedish cohort (**B**,**D**,**F**). Abbreviations: PDGFR, platelet-derived growth factor receptor; SCC, squamous cell carcinoma; ADC, adenocarcinoma.

**Figure 3:**
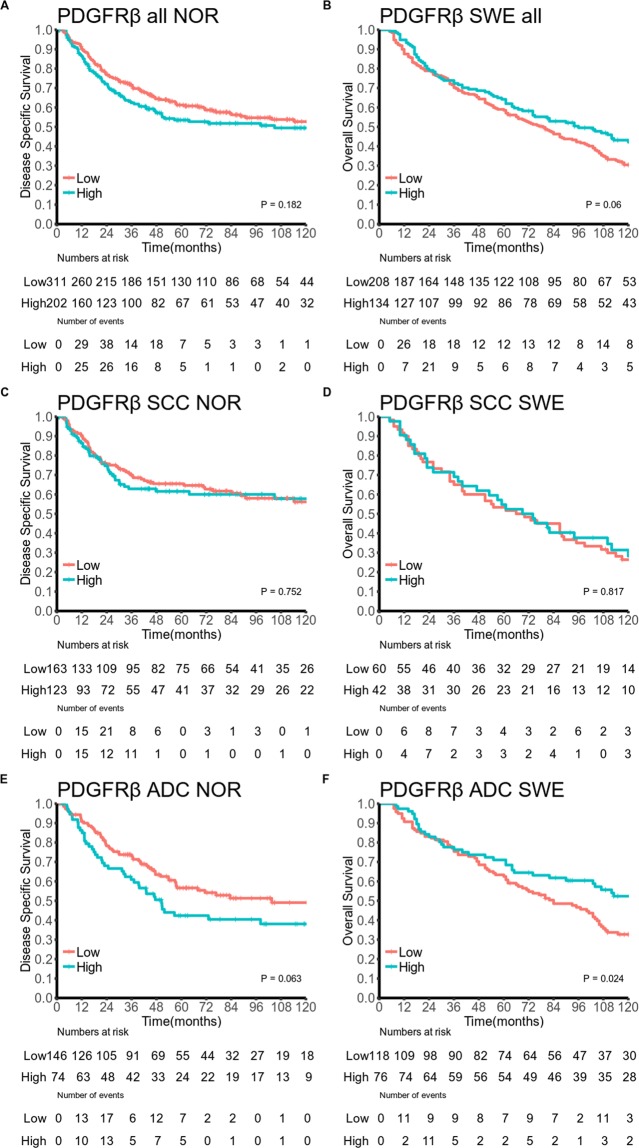
Survival curves for PDGFRβ expression in the overall cohorts and in the SCC and ADC subgroups for the Norwegian cohort (**A**,**C**,**E**) and the Swedish cohort (**B**,**D**,**F**). Abbreviations: PDGFR, platelet-derived growth factor receptor; SCC, squamous cell carcinoma; ADC, adenocarcinoma.

Table 3 and Figs 2 and 3 summarize the univariate survival analyses of marker expression. In the overall Norwegian cohort neither PDGFRα, nor PDGFRβ, were significantly associated with DSS. In the overall Swedish cohort high expression of PDGFRα (HR = 0.66, 95% CI 0.5–0.87, P = 0.006) was associated with increased OS.

**Table 3:** PDGFR-α, PDGFR-β as predictors of (A) disease-specific survival in a Norwegian cohort of 553 stage I-IIIB NSCLC patients (307 and 239 in the SCC and ADC subgroups respectively) and (B) overall survival in a Swedish cohort of 367 stage I NSCLC patients (109 and 209 in SCC and ADC subgroups respectively, log-rank test)

	(A) Norwegian cohort					(B) Swedish cohort				
	N(%)	5 Year	Median	HR(95%CI)	P	N(%)	5 Year	Median	HR (95%CI)	P
	Overall cohort									
PDGFR-α					0.124					**0.006**
Low	366 (66)	57	127	1		232 (63)	57	74	1	
High	152 (27)	65	235	0.78 (0.58–1.05)		113 (31)	70	104	0.66 (0.5–0.87)	
Missing	35 (6)					22 (6)				
PDGFR-β					0.182					0.060
Low	311 (56)	61	190	1		208 (57)	59	79	1	
High	202 (37)	54	105	1.21 (0.91–1.6)		134 (37)	64	96	0.77 (0.59–1)	
Missing	40 (7)					25 (7)				
	Squamous cell carcinoma									
PDGFR-α					**0.020**					**0.003**
Low	204 (66)	60	NA	1		70 (64)	46	54	1	
High	85 (28)	76	235	0.57 (0.37–0.87)		32 (29)	75	NA	0.43 (0.27–0.7)	
Missing	18 (6)					7 (6)				
PDGFR-β					0.752					0.817
Low	163 (53)	65	NA	1		60 (55)	53	68	1	
High	123 (40)	62	235	1.07 (0.72–1.59)		42 (39)	55	72	0.95 (0.59–1.51)	
Missing	21 (7)					7 (6)				
	Adenocarcinoma									
PDGFR-α					0.962					**0.038**
Low	158 (66)	53	73	1		134 (64)	64	91	1	
High	64 (27)	53	98	1.01 (0.65–1.56)		64 (31)	72	NA	0.64 (0.44–0.95)	
Missing	17 (7)					11 (5)				
PDGFR-β					0.063					**0.024**
Low	146 (61)	57	104	1		118 (56)	63	84	1	
High	74 (31)	42	50	1.45 (0.96–2.19)		76 (36)	71	NA	0.64 (0.44–0.93)	
Missing	19 (8)					15 (7)				

Abbreviations: PDGFR, platelet-derived growth factor receptor; NSCLC, non-small cell lung cancer; SCC, squamous cell carcinoma; ADC, adenocarcinoma.

In SCC patients, increased expression of PDGFRα was associated with increased DSS in the Norwegian cohort (HR = 0.57, 95% CI 0.37–0.87, P = 0.020) and OS in the Swedish cohort (HR = 0.43, 95% CI 0.27–0.70), P = 0.003). In the Norwegian cohort, the association was present through all pStages although only significant in pStage II and III (data not shown). In ADC patients, increased expression of PDGFRα (HR = 0.64, 95% CI 0.44–0.95, P = 0.038) and PDGFRβ (HR = 0.64, 95% CI 0.44–0.93, P = 0.024) were associated with increased OS in the Swedish cohort. PDGFRβ showed a non-significant association with decreased DSS in the Norwegian cohort (HR = 1.45, 95% CI 0.96–2.19, P = 0.063)

#### Multi-variable analyses

Table 4 summarizes the multi-variable models for DSS and OS in both cohorts (models 1 and 4) and in the SCC and ADC subgroups (models 2, 3, 5 and 6).

**Table 4:** Multivariable analysis of clinicopathological variables, PDGFRα and PDGFRβ in the overall cohorts (Models 1 and 4) and in the SCC and ADC subgroup (Models 2,3,5 and 6).

	All patients		SCC		ADC	
	Norwegian cohort					
	Model 1		Model 2		Model 3	
	HR (95% CI)	P	HR (95% CI)	P	HR (95% CI)	P
Gender						
Female	1				1	
Male	1.46 (1.06–1.99)	**0.019**			1.46 (0.98–2.19)	0.063
Histology						
SCC	1					
ADC	1.4 (1.05–1.88)	0.024				
NOS	0.54 (0.13–2.27)	0.404				
Pstage						
I	1		1		1	
II	1.57 (1.1–2.24)	**0.014**	1.49 (0.89–2.51)	0.128	1.88 (1.15–3.08)	**0.012**
III	3.88 (2.72–5.54)	**<0.001**	6.1 (3.64–10.24)	**<0.001**	3.85 (2.35–6.29)	**<0.001**
Differentiation						
Poor	1				1	
Moderate	0.91 (0.67–1.22)	0.518			1.04 (0.68–1.6)	0.848
Well	0.56 (0.34–0.92)	0.022			0.53 (0.29–0.99)	0.047
Vascular invasion						
No	1		1			
Yes	1.63 (1.15–2.31)	**0.006**	1.7 (1.07–2.69)	**0.025**		
PDGFRα						
Low	1		1			
High	0.66 (0.47–0.93)	**0.016**	0.37 (0.21–0.63)	**<0.001**		
PDGFRβ						
Low	1		1		1	
High	1.44 (1.06–1.94)	**0.020**	1.51 (0.97–2.33)	0.067	1.48 (1–2.21)	0.053
	Swedish cohort					
	Model 4		Model 5		Model 6	
	HR (95% CI)	P	HR (95% CI)	P	HR (95% CI)	P
Age	1.02 (1.01–1.04)	**0.005**	1.04 (1.01–1.08)	**0.013**		
Gender						
Female	1				1	
Male	1.53 (1.16–2)	**0.002**			1.62 (1.12–2.34)	**0.010**
PDGFRα						
Low	1		1			
High	0.67 (0.5–0.91)	**0.010**	0.39 (0.22–0.69)	**0.001**		
PDGFRβ						
Low						
High					0.62 (0.42–0.92)	**0.020**

Abbreviations: PDGFR, platelet-derived growth factor receptor; NSCLC, non-small cell lung cancer; SCC, squamous cell carcinoma; ADC, adenocarcinoma; NOS, not otherwise specified.

In the overall Norwegian cohort, PDGFRα was an independent predictor of increased DSS in both the overall cohort (adjusted HR = 0.66, 95% CI 0.47–0.93, P = 0.016) and the SCC subgroup (adjusted HR = 0.37, 95% CI 0.21–0.63, P < 0.001). Likewise, in the Swedish cohort, PDGFRα was an independent predictor of increased OS both in the overall cohort (adjusted HR = 0.67, 95% CI 0.50–0.91, P = 0.010) and in the SCC subgroup (adjusted HR = 0.39, 95% CI 0.22–0.69, P = 0.001).

In the overall Norwegian cohort, PDGFRβ was an independent predictor of poor DSS (adjusted HR = 1.44, 95% CI 1.06–1.94, P = 0.020), while non-significant correlations were noted in the SCC (P = 0.067) and ADC (P = 0.053) subgroups. In the Swedish cohort, PDGFRβ was an independent predictor of increased OS in the ADC subgroup (adjusted HR = 0.62, 95% CI 0.42–0.92, P = 0.020).

#### Co-expressions

In the Norwegian cohort, significant correlations between the expression of PDGFRα and -β was observed. A similar trend was observed in the Swedish cohort. On this basis, co-expressions were explored (supplementary Table 1 and Supplementary Fig. 1). In both cohorts, patients presenting PDGFRα+/β+ were among the groups with highest survival. In the Norwegian cohort, patients presenting PDGFRα−/β+ exhibited inferior survival according to increasing stage (Supplementary Fig. 2). Multi-variable analyses of co-expressions in the Norwegian cohort corrected by pStage confirmed that the expression pattern PDGFRα−/β+ (HR 1.74 95% CI 1.25–2.42, P = 0.001) was associated with adverse survival.

## Discussion

This study confirms that high stromal expression of PDGFRα is an independent marker associated with a favorable prognosis in NSCLC patients. Further, co-expression analyses indicates that relative expression of PDGFRs impact on survival in a pStage and histotype specific manner.

NSCLC represent a morphological and clinical heterogeneous cancer type, with adenocarcinomas and squamous cell lung cancer as the predominant histological subtypes. Earlier studies on the prognostic relevance of PDGFRs in NSCLC are scarce and inconclusive. In two previous studies from our group, including 335 resected specimens from NSCLC patients, high stromal expression of PDGFRα was associated with longer survival in univariate analyses, whereas stromal PDGFRβ did not show any prognostic value^11,21^. Interestingly, stromal PDGFRβ was associated with locoregional disease^21^. In a third study analyzing the prognostic relevance of twelve stromal markers including PDGFRβ, no prognostic associations were found for this marker as observed in our study^22^.

In the present study, high stromal expression of PDGFRα was an independent marker of increased survival in the overall cohort and in the SCC subgroups of both the Norwegian and Swedish cohorts. However, in univariate analysis of the overall Norwegian cohort, the expression of PDGFRα did not reach statistical significance. Nevertheless, we believe that these robust findings, from multivariable analyses of two cohorts, confirm our previous results of PDGFRα as a strong prognosticator of increased survival in NSCLC patients^11,21^. Intriguingly, PDGFRβ was an independent marker of decreased DSS in the overall Norwegian cohort (Table 4, Fig. 3). This finding, however, could not be confirmed in the Swedish cohort. On the contrary, PDGFRβ was an independent predictor of increased OS in Swedish ADC patients. No final conclusion on the prognostic impact of PDGFRβ in NSCLC can be drawn based on these data. The findings may be due to false positive results or functional aspects of PDGFRβ positive cells differing according to pStage and/or histological subtype. In addition, Further, co-expression analyses indicate that the relative expression of PDGFRs are pivotal in a prognostic setting and that their prognostic impact differs with changing pStage and histological entity. However, the current study was not powered to investigate PDGFRs in all pStages stratified by histology.

The underlying mechanisms behind the observed associations are likely complex and multi-factorial. PDGF signaling, known to be essential in embryonic development, is also involved in various pathophysiological processes including fibrosis, atherosclerosis and tumorigenesis^23^. In epithelial tumors, PDGF is thought to act mainly in a paracrine fashion, affecting stromal cells such as fibroblasts and pericytes^24^. Cancer-associated fibroblasts, or CAFs, represents a widespread cell type in NSCLC, and can facilitate growth-suppressing or growth-promoting signals depending on the context. A number of studies have demonstrated that ligand-mediated activation of PDGFR signaling induces recruitment, proliferation and differentiation of mesenchymal cells into tumors^23,25^. PDGF signaling on CAFs may also impact extra-cellular matrix deposition and tissue stiffness. In animal models, inhibition of PDGFR signaling decreases interstitial fluid pressure and increases intratumoral drug uptake^26,27^. Of note, in a recent study by us comparing tissue expression of different stromal markers in the same NSCLC cohort used here, we did not observed correlations between PDGFRs expression and collagen deposition^20^. Furthermore, PDGF-stimulated fibroblasts have been shown to produce factors involved in the invasion and metastasis of colorectal cancer cells^28^, and a similar mechanism has been proposed for induction of epithelial to mesenchymal transition in liver cancer and metastatic prostate cancer^29,30^.

The PDGF/PDGFR axis plays a fundamental role in the regulation of tumor angiogenesis and lymphangiogenesis. A large set of studies have demonstrated the importance of PDGFRβ-positive perivascular cells, or pericytes, in tumor vessel stabilization. Experimental studies in different animal cancer models have shown that reduction of pericyte recruitment, through interference with the PDGFRβ signaling in pericytes, negatively affects tumor angiogenesis and also reduces tumor growth^31,32^. However, other studies, in different cancer models, have demonstrated that pericyte depletion through interference with PDGFRβ signaling can favor tumor growth^33,34^. This indicates that activation of PDGF signaling components in angiogenesis and lymphangiogenesis, is likely context-dependent and seems to vary among tumor types and stages. In the present study, PDGFRβ expression was not restricted to perivascular cells and it remains to be studied if the presence of PDGFRβ-positive pericytes has an impact on the survival of NSCLC patients.

A main concern of the current study is the use of TMAs, which do not allow assessment of zonal expression of the receptors in spatially restricted regions of the tumor, such as the invasive front and the perivascular areas. However, with the aim of validating the TMA approach, we also performed PDGFRα and β immunostaining and scoring on whole tissue slides (WTS) from 35 patients in the Norwegian cohort, including the two histological subgroups and patients from stage I and stage III. Interestingly, intensity and density in WTSs were not significantly correlated to TMA (data not shown). This finding may be due to small differences in staining, inter- and intrarater variability or tumor heterogeneity.

Ligand binding to PDGFRs leads to PDGF receptor dimerization, phosphorylation and activation. The α- and the β-receptors are structurally related, both receptors are featured by an intracellular tyrosine-kinase domain, and both receptors transduce overlapping although not identical cellular signals. In spite of their well described similarities, their significance as prognostic markers appears in most instances opposed. It remains uncertain why the α-receptor associates often with good prognosis while the β-receptor correlates with poor prognosis in many common solid tumors. A potential explanation may rely not on the receptors *per se* but on the cells expressing the receptors. Thus, according to our results, it is possible that PDGFRα expression reflects a growth restraining fibroblast population. Unfortunately, analyses of receptor co-expression in the same slides did not work out well in our system and could not be compared in this study. This latter finding may be due to over-expression of either PDGFRα or −β. Further studies should aim at confirming our results in different cohorts and ideally with different antibodies. However, a recent analysis of breast DCIS associated a PDGFRα+/β− fibroblast phenotype in stroma with favorable prognosis^35^. This publication further corroborates that the two PDGFRs are independently expressed and may have different functions and/or mark functionally distinct fibroblasts.

In conclusion, the presented results indicate that high stromal expression of PDGFRα is a strong and independent predictor of longer survival for pStage I-III NSCLC patients. The association is particularly strong in the SCC histological subgroup. Further, even though the prognostic impact of PDGFRβ expression differs between the two cohorts, co-expression analyses indicates that the relative expression of PDGFRs impact on survival in a pStage and histotype specific manner. These findings should be emphasized when considering PDGFR-targeted therapy for NSCLC patients.

## Supplementary information

Supplementary information

## Data Availability

The datasets generated during and/or analysed during the current study are available from the corresponding author on reasonable request.
