# Supplementary material for: Differential prognostic impact of platelet-derived growth factor receptor expression in NSCLC
Source: Sci Rep. 2019 Jul 15;9:10163. doi: 10.1038/s41598-019-46510-3 (PMC6629689; doi:10.1038/s41598-019-46510-3)

Differential prognostic impact of platelet-derived growth factor receptor expression in NSCLC

Thomas Karsten Kilvaer[1, 2]

Mehrdad Rakaee[2, 3]

Turid Hellevik[1, 2]

Jørg Vik[2]

Luigi De Petris[4]

Tom Donnem[1, 2]

Carina Strell[4]

Arne Ostman[4]

Lill-Tove Rasmussen Busund[3, 5]

Inigo Martinez-Zubiaurre[2]

1 Department of Oncology, University Hospital of North Norway, Tromso, Norway
2 Institute of Clinical Medicine, UiT The Arctic University of Norway, Tromso, Norway
3 Institute of Medical Biology, UiT The Arctic University of Norway, Tromso, Norway
4 Department of Oncology-Pathology Cancer Center Karolinska, Karolinska Institutet, Stockholm Sweden
5 Department of Clinical Pathology, University Hospital of North Norway, Tromso, Norway

Corresponding author and reprints:

Thomas K. Kilvaer, MD PhD

Department of Oncology, University Hospital of North Norway

9038 Tromso, Norway

Telephone: +47 776 26 765/+47 905 24 635

Fax: +47 77626779

E-mail: kilvaer@gmail.com kilvaer@gmail.com

Supplementary table 1: Co-expression ofPDGFR-α and PDGFR-β as predictors of A) disease-specific survival in a Norwegian cohort of 553 stage I-IIIB NSCLC patients (307 and 239 in the SCC and ADC subgroups respectively) and B) overall survival in a Swedish cohort of 367 stage I NSCLC patients (109 and 209 in SCC and ADC subgroups respectively, log-rank test)

	A) Norwegian cohort					B) Swedish cohort				
	N(%)	5 Year	Median	HR(95%CI)	P	N(%)	5 Year	Median	HR(95%CI)	P
Overall cohort										
					0.012					0.018
α-/β-	247(45)	62	190	1		157(43)	56	76	1	
α-/β+	112(20)	43	47	1.57(1.09-2.26)		72(20)	58	68	1.01(0.71-1.44)	
α+/β-	62(11)	59	NA	1.03(0.67-1.59)		49(13)	67	87	0.9(0.6-1.34)	
α+/β+	88(16)	68	235	0.81(0.55-1.18)		61(17)	70	NA	0.52(0.37-0.74)	
Missing	44(8)					28(8)				
Squamous cell carcinoma										
					0.059					0.068
α-/β-	133(43)	64	NA	1		44(40)	45	52	1	
α-/β+	67(22)	50	64	1.38(0.82-2.33)		26(24)	46	58	1.04(0.57-1.91)	
α+/β-	29(9)	74	NA	0.69(0.36-1.32)		14(13)	79	99	0.45(0.23-0.87)	
α+/β+	54(18)	76	235	0.61(0.36-1.02)		16(15)	69	112	0.51(0.27-0.99)	
Missing	24(8)					9(8)				
Adenocarcinoma										
					0.051					0.026
α-/β-	113(47)	59	190	1		94(45)	62	84	1	
α-/β+	42(18)	33	41	1.89(1.12-3.19)		37(18)	68	103	0.92(0.56-1.53)	
α+/β-	32(13)	48	57	1.33(0.74-2.39)		24(11)	66	80	1.06(0.58-1.94)	
α+/β+	32(13)	57	98	1.08(0.61-1.93)		39(19)	74	NA	0.42(0.26-0.67)	
Missing	20(8)					15(7)				

Abbreviations: PDGFR, platelet-derived growth factor receptor; NSCLC, non-small cell lung cancer; SCC, squamous cell carcinoma; ADC, adenocarcinoma.

Supplementary figure 1: Survival curves for the co-expression of PDGFRα and PDGFRβ in the overall cohorts and in the SCC and ADC subgroups for the Norwegian cohort (A, C and E) and the Swedish cohort (B, D and F)

Abbreviations: PDGFR, platelet-derived growth factor receptor; SCC, squamous cell carcinoma; ADC, adenocarcinoma.

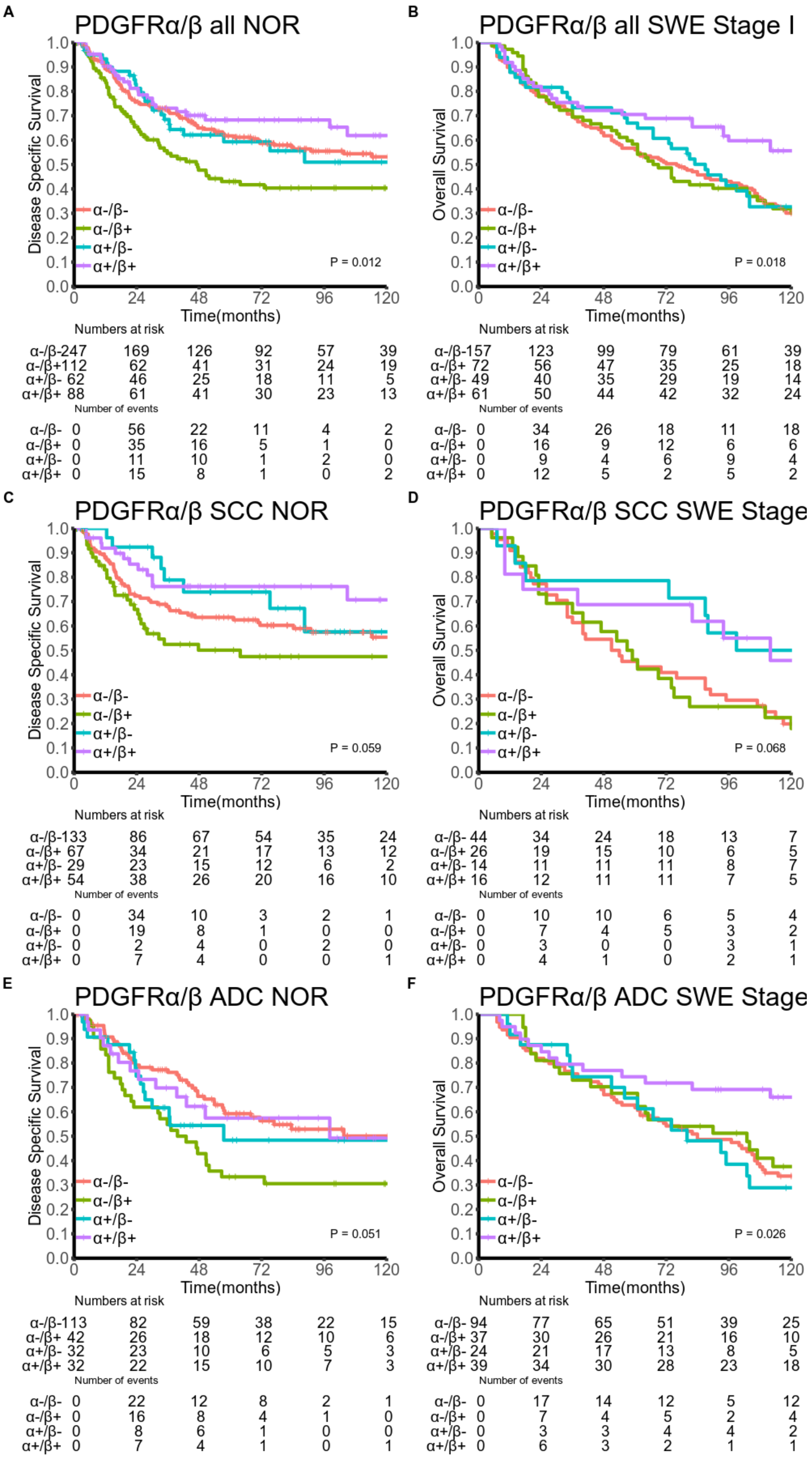

Supplementary figure 2: Survival curves for the co-expression of PDGFRα and PDGFRβ in Norwegian cohort according to increasing pStage

Abbreviations: PDGFR, platelet-derived growth factor receptor; SCC, squamous cell carcinoma; ADC, adenocarcinoma.

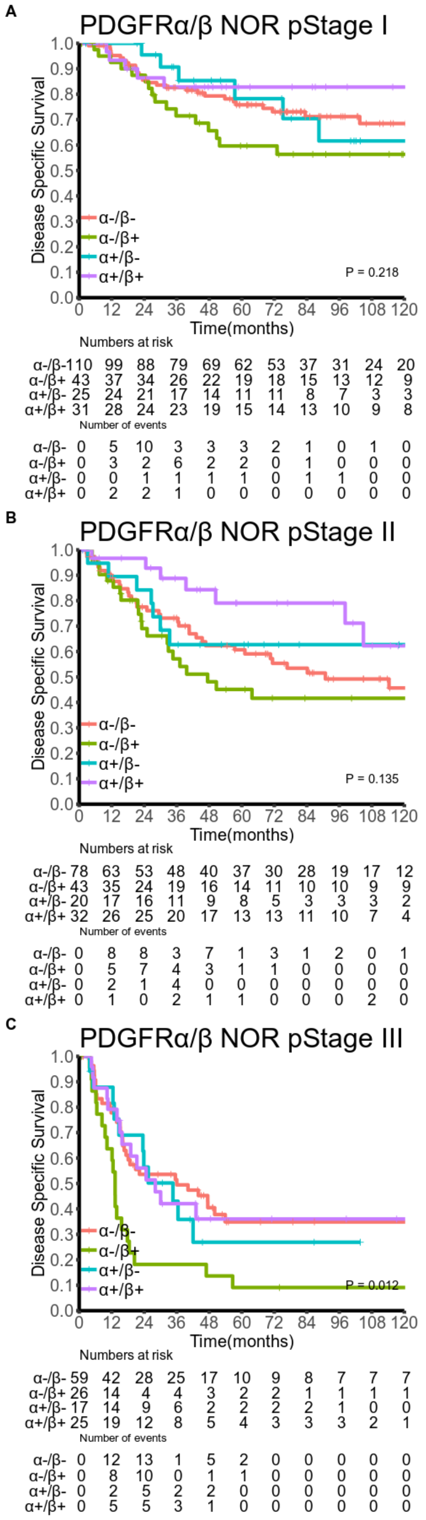

Supplement: Supplementary file 1 — Supplementary information [file 41598_2019_46510_MOESM1_ESM.pdf]
